# Riesgos reputacionales derivados de la presencia de las enfermeras en redes sociales y propuesta de acción. El caso *Vall d’Hebron*

**DOI:** 10.23938/ASSN.1095

**Published:** 2025-01-15

**Authors:** Hildegart González-Luis, Ana Azurmendia, Blanca Basanta-Vázquez, Francesc Pujol

**Affiliations:** 1 University of Navarra School of Nursing Department of Community, Maternity and Pediatric Nursing Pamplona Spain; 2 George Washington University School of Nursing Center for Health Policy and Media Engagement Washington USA; 3 University of Navarra Innovation for a Person-Centred Care Research Group (ICCP-UNAV) Pamplona Spain; 4 Navarra’s Health Research Institute (IdiSNA) Pamplona Spain; 5 University of Navarra School of Communication Department of Public Communication Pamplona Spain; 6 University of Navarra DigitalUnav Center for Internet Studies and Digital Life Pamplona Spain; 7 University of Navarra School of Communication Pamplona Spain; 8 University of Navarra School of Economics Department of Economics Pamplona Spain

**Keywords:** Enfermería, Redes Sociales, Percepción, Oratoria, Guía, Nursing, Perception, Social Media, Speech, Guideline

## Abstract

**Fundamento:**

Una enfermera española publicó en *TikTok* un vídeo criticando el requisito de disponer del nivel C1 de catalán para obtener plaza en el sistema sanitario público de Cataluña (España), que se viralizó en redes sociales (RRSS) y provocó la reacción de usuarios, políticos e instituciones de enfermería. El objetivo es evaluar el daño reputacional que sufrió la enfermera, su hospital y la profesión, y proponer líneas de acción para situaciones similares.

**Metodología:**

Diseño de metodología mixta secuencial exploratoria para analizar el contenido del vídeo; el impacto que su contenido generó en prensa y RRSS, y la gestión comunicativa de la crisis realizada por el hospital implicado y los colegios de enfermería.

**Resultados:**

La enfermera manifestó su opinión amparada por el derecho a la libertad de expresión, pero el lenguaje empleado, vestir el uniforme de un hospital concreto y la crítica al idioma fueron los factores que dañaron su reputación al difundirse por la prensa y en la conversación de *Twitter/X*La reputación del hospital se vio afectada por ser identificable la enfermera como trabajadora del mismo, mientras que la del sector enfermero apenas se vio perjudicada gracias al amplio abanico de fuentes informativas que lo identificaron como un suceso aislado. Se proponen dos líneas de acción: la creación de un marco autorregulatorio seguro y de un plan de formación en RRSS lideradas por centros sanitarios, asociaciones y facultades de enfermería.

**Conclusión:**

La reputación de la enfermera y del hospital resultaron dañadas. Se aporta un modelo metodológico para analizar situaciones similares.

## Abstract (en)

**Background:**

A Spanish nurse posted a video on TikTok criticizing the requirement of of a certain level of Catalan to work in the public health system of Catalonia (Spain). The video went viral, eliciting reactions from social media users, politicians, and professional nursing organizations. This study aims to evaluate the reputational damage suffered by the nurse, her hospital, and the nursing profession, with the goal of proposing actions to prevent, avoid, or manage similar situations.

**Methodology:**

A sequential exploratory mixed-methods design was used to analyse the content of the video, its impact on the press and social media, and the management of the communication crisis by the hospital and nursing organizations involved.

**Results:**

The nurse exercised her right to freedom of expression. However, her choice to convey the message, wearing the uniform that identified her as a staff member of a specific hospital, and her criticism of the language level requirement, led to reputational damage after the video spread via the press and Twitter/X. The hospital’s reputation was affected due to the nurse being identified as one of its employees. In contrast, the reputation of the nursing profession was not significantly harmed, as multiple information sources suggested the incident was isolated. The creation of a safe, self-regulatory framework and training in social media and communication management are proposed as measures for healthcare centres, nursing organizations, and academic institutions to implement.

**Conclusions:**

The reputations of both the nurse and her hospital were impacted. A methodological model for analysing similar situations is proposed.

## INTRODUCCIÓN

En 2005 la Organización Mundial de la Salud en su 58ª Asamblea instó a los estados miembros a elaborar un plan estratégico a largo plazo para concebir e implantar servicios de cibersalud^1^La pandemia de COVID-19 reforzó la necesidad de ampliar los servicios *online* y, tal y como reflejó en 2022 la encuesta sobre Equipamiento y Uso de Tecnologías de Información y Comunicación (TIC) en los hogares españoles, el 77% de las personas de 16 a 74 años había realizado en los últimos tres meses alguna actividad *online* relacionada con la salud y, respecto al año anterior, el acceso a archivos personales sanitarios aumentó en 22,5 puntos y el acceso a otros servicios de salud en 14,4^2^.

Ante esta realidad, la presencia proactiva de las enfermeras en el ámbito *online* es cada día más necesaria para facilitar la consecución de la amplia variedad de funciones y servicios que aportan al conjunto de la sociedad^3^^-^^5^Diversos estudios muestran la utilidad de la participación enfermera en redes sociales para promover la salud de la población y combatir la desinformación^6^^,^^7^, compartir conocimientos y aumentar la evidencia científica enfermera^8^^,^^9^, formar a las nuevas generaciones^10^^,^^11^, incrementar el liderazgo^12^^,^^13^, lograr y aumentar su influencia en políticas sanitarias^14^^,^^15^, e incluso mejorar la imagen de la enfermería^16^^,^^17^.

Los medios de comunicación son uno de los agentes más influyentes en la imagen que la sociedad tiene de las enfermeras. Diversas revisiones de la literatura concluyen que la imagen que difunden no refleja adecuadamente la identidad de esta profesión^18^^-^^23^Esta imagen distorsionada perjudica la autopercepción que tienen las enfermeras de su profesión^24^^,^^25^, aumenta la intención de abandonar su trabajo y disminuye la demanda de estudiantes que quieren cursar el grado que les habilita para ejercer^26^^-^^29^Las redes sociales han sido propuestas como canales idóneos para que sean las propias enfermeras las que, evitando la mediatización de los profesionales de la comunicación, expongan a la sociedad su aportación en los sistemas sanitarios del siglo XXI^16^^,^^17^.

A pesar de los beneficios de la presencia proactiva de las enfermeras en redes sociales, diversos autores advierten de los riesgos que implica para la reputación personal, institucional (centros sanitarios en los que trabajan) y profesional (conjunto de profesionales)^30^^,^^31^, por lo que la perspectiva deontológica y jurídica (normas que protegen la reputación) que regula la actuación de los profesionales de la salud también es relevante para comprender esta cuestión. La gestión de crisis reputacionales ha alcanzado una nueva dimensión con la expansión masiva del uso de redes sociales.

La Teoría de la Comunicación de Crisis Situacional (SCCT), propuesta en 2002^32^ y desarrollada en 2007^33^, sigue siendo un marco de referencia en este campo. Abarca tanto la gestión y respuesta a eventos de crisis como un proceso completo de planificación de escenarios de crisis, con el objetivo de prevenir la crisis y, en caso de que ocurra, mitigar su daño mediante el diseño de protocolos y programas de formación.

En las redes sociales, todos los usuarios son potenciales creadores de contenido, y cualquier contenido puede convertirse en viral rápidamente por las dinámicas de difusión. Así, cualquier empleado, cliente o usuario puede convertirse en agente activador de crisis corporativas de reputación, incluso si cuenta con muy pocos seguidores^34^En redes sociales, la rapidez de respuesta se ha vuelto crítica para gestionar efectivamente las crisis^35^Aunque la estructura fundamental de la SCCT sigue siendo válida, la presencia generalizada de redes sociales ha desembocado en la adaptación de propuestas tanto para la gestión de crisis como para su planificación^36^^,^^37^.

El 2 de marzo de 2023, tres enfermeras del hospital catalán *Vall d’Hebron* protagonizaron en *TikTok* un vídeo de 1 min y 26 s que se hizo viral alcanzando más de 25 millones de visualizaciones^38^En él, una de ellas criticó que las personas candidatas a *sacarse* una oposición de enfermería en Cataluña deben acreditar el nivel C1 de catalán. El vídeo se difundió también en otras redes sociales, como *Twitter* (ahora *X*) y en medios de comunicación, convirtiendo la crítica de la enfermera en noticia.

A primera vista, el motivo por el que se viralizó el vídeo se debe a que la enfermera realizó una crítica en un tema sobre el que hay disparidad de opiniones y posturas en la sociedad española: la idoneidad de requerir un título que certifique un nivel de conocimiento de una lengua cooficial en determinadas comunidades autónomas para poder trabajar con una plaza en propiedad en la administración pública de dicho territorio.

Este vídeo de *TikTok* en el que la enfermera ejerció su derecho a la libertad de expresión provocó una conversación ruidosa en redes sociales en la que fue atacada por oponerse a obtener el nivel C1 de catalán.

El objetivo de este estudio es evaluar el daño reputacional sufrido por la enfermera, el hospital en el que trabajaba y la profesión de enfermería, causado por la publicación de noticias en la prensa y *posts* en redes sociales sobre este vídeo. Con ello se pretende realizar una propuesta que contribuya a prevenir, evitar o gestionar este tipo de situaciones.

## MATERIAL Y MÉTODOS

Debido a la novedad y naturaleza del objeto de estudio, se decidió que el diseño más apropiado para abordarlo era una metodología mixta secuencial exploratoria que a través de distintos métodos posibilitara reunir datos cualitativos y cuantitativos que, tras su triangulación-integración, permitieran la extracción de interpretaciones^39^^,^^40^.

**Figura 1:**
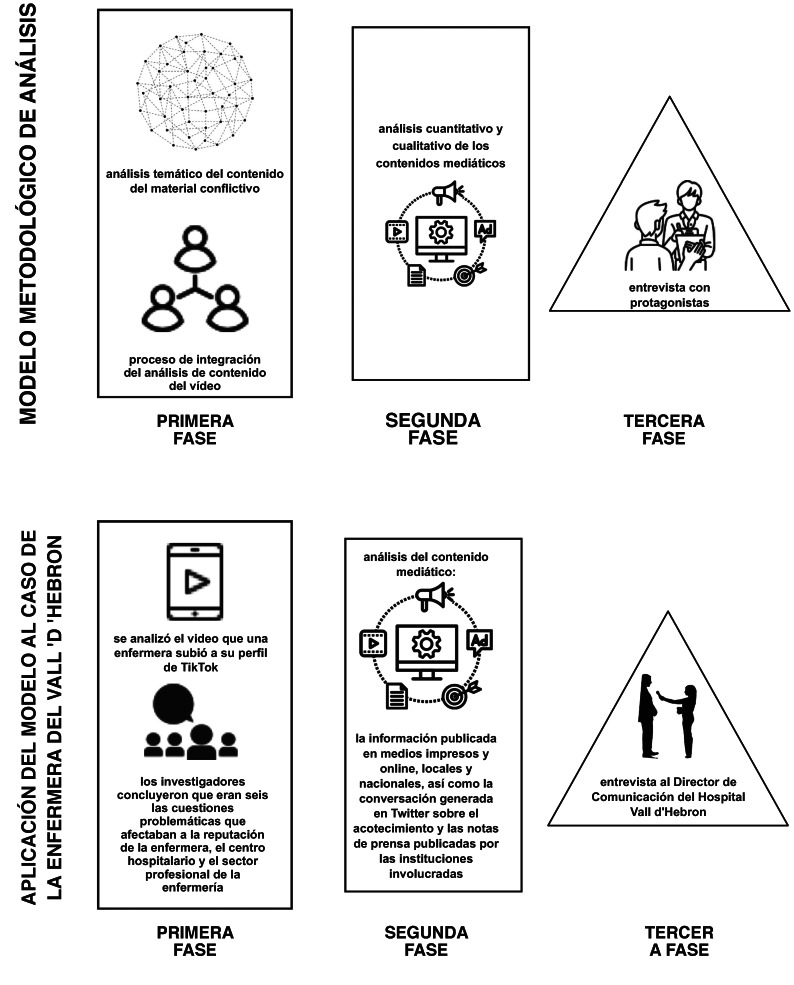
Modelo metodológico de análisis y aplicación al caso de estudio

Para realizar el estudio se recurrió a cinco metodologías, aplicadas de manera secuencial en tres fases (Fig. 1) que, dada la naturaleza de las muestras, no requirieron la aprobación de un comité de ética^41^.

1. *Análisis temático del contenido del vídeo*^41^ para identificar y explicar, mediante la revisión de las normativas jurídicas y éticas que rigen la práctica de la enfermería, los aspectos problemáticos presentes en el vídeo que podían ser dañinos para la reputación de la enfermera, del sector enfermero y del hospital.

Fue realizado por tres miembros del equipo investigador (profesora de Derecho de la Comunicación, periodista, Máster en Gestión de Empresas de Comunicación y profesora de Enfermería) con conocimiento de leyes y códigos deontológicos vigentes, además de aspectos de comunicación institucional, en particular de comunicación institucional referida a la enfermería. Se garantizó un análisis multidisciplinar para identificar las cuestiones problemáticas del vídeo por los que el contenido difundido podía ser evaluado/juzgado.

Cada una de ellas elaboró un listado que fue puesto en común y discutido para integrar los resultados obtenidos, que permitieron diseñar la *Guía para el análisis de las noticias, notas de prensa y post en Twitter* (Tabla 2), que permitió obtener evidencia sobre el eco de las cuestiones identificadas como problemáticas en noticias, *tweets* y notas de prensa, además de las fuentes de los periodistas y otros actores de la conversación de *Twitter*.

2. *Análisis de contenido cualitativo de las noticias* para conocer 1) si los aspectos problemáticos fueron difundidos y criticados, afectando a la reputación de los tres actores mencionados; 2) cuáles fueron las temáticas que hilaron la narrativa informativa e institucional y la conversación en redes sociales; y 3) quiénes fueron las fuentes informativas principales utilizadas por los periodistas y los actores que intervinieron en la conversación, y si lo aportado por ellos perjudicó la imagen/reputación de los tres actores.

A través de la base de datos *mynews*, que recopila la información publicada en 1.585 medios de comunicación españoles impresos y *online*, se realizó una búsqueda avanzada para identificar las noticias sobre el caso. Los criterios de selección de las noticias fueron: informaciones difundidas en medios de España escritos en español desde el 2 de marzo hasta el 17 de mayo de 2023 que contuvieran la combinación de palabras: “*Vall d’Hebron*” AND “*Tiktok*” AND “enfermera”.

Las tres investigadoras aplicaron el instrumento diseñado (Tabla 2) para extraer la información sobre un 10% de la muestra a fin de evaluar el grado de acuerdo entre codificadoras; una vez confirmado, cada una extrajo la información de un tercio de la muestra restante. Todos los datos extraídos fueron analizados e interpretados conjuntamente por las tres investigadoras.

3. *Búsqueda de la conversación generada en Twitter* para entender cómo fue la conversación en redes sociales, quiénes participaron y en qué se puso el foco.

Se realizó una búsqueda con los términos “enfermera” y “*Vall*”, y se extrajeron aquellos tuits visualizados en pantalla por al menos 500 usuarios. Una vez creada la base de datos de texto (mensajes de *Twitter*), el análisis sintáctico de la conversación se realizó con la herramienta *Graphex*, que con sus funcionalidades permite el análisis de relaciones y estructuras dentro de redes complejas. Se pudo estudiar cómo sucedió la conversación en *Twitter* durante la primera semana que duró el caso analizado, así como identificar las comunidades para comprender quiénes participan en la conversación, de qué modo y sobre qué ponían el foco del acontecimiento. Esto permite la categorización cuantitativa de la conversación mediante la visualización de su estructura sintáctica: 1) cada nodo representa un tuit de la conversación; 2) la cercanía entre nodos representa la similitud de redacción entre los mensajes; 3) cada tuit se agrupa en diferentes categorías temáticas (barras de colores en sentido horizontal) en función de los términos más repetidos en cada mensaje.

4. *Análisis de dos notas de prensa remitidas de manera independiente por dos instituciones afectadas*, el *Col·legi d’Infermeres* de Barcelona (COIB) y el Excelentísimo Colegio Oficial de Enfermería de Sevilla (ECOES).

Fue realizado por las tres investigadoras con la guía obtenida con la primera metodología.

5. *Entrevista al Director de Comunicación, Estrategia Corporativa y Atención Ciudadana del Campus Vall d’Hebron, Francisco García Morales*, con la finalidad de obtener información de primera mano sobre cómo gestionó la crisis el hospital en el que trabajaba la enfermera.

Se consideró oportuna esta metodología tras confirmar que el hospital *Vall d’Hebron* no había remitido ninguna nota de prensa oficial. También se intentó mantener una entrevista con la enfermera protagonista del caso, sin éxito.

## RESULTADOS

El *análisis temático del contenido del vídeo* permitió identificar seis cuestiones problemáticas en el vídeo (Tabla 1) que permitieron diseñar la *Guía para el análisis de las noticias, notas de prensa y post en Twitter* (Tabla 2).

**Tabla 1:** Seis cuestiones problemáticas del vídeo

Cuestión problemática del vídeo	Ley o código que podría incumplir	
1	El no uso de la mascarilla por parte una enfermera en un hospital	Real Decreto 286/2022, de 19 de abril.
2	Vídeo grabado por una enfermera en horario laboral, dentro de un hospital	Ley 44/2003, de 21 de noviembre, de Ordenación de las Profesiones Sanitarias, art. 4 y 5.
3	Las tres enfermeras llevan puesto el uniforme del Instituto Catalán de Salud	Ley 55/2003, del Estatuto Marco del personal estatutario de los servicios de salud.
4	En el video se ve una pantalla de ordenador del hospital que podía contener datos confidenciales de pacientes	Ley Orgánica 3/2018, de Protección de Datos y de Garantía de los Derechos Digitales.
5	Aspectos formales y de contenido de la comunicación empleada por la enfermera	Código de Ética del CIE para las enfermeras.
6	Crítica a que se exija el C1 para poder obtener una plaza pública en propiedad	Código de Ética del CIE para las enfermeras.

**Tabla 2:** Guía con ítems identificados para el análisis de las noticias, notas de prensa y *posts* en *Twitter*

Item	Preguntas para analizar los contenidos del vídeo
1	¿Se critica o menciona el no uso de la mascarilla por parte de las enfermeras?
2	¿Se menciona o critica la pérdida de tiempo de las enfermeras en horario laboral?
3	¿Se menciona o critica que llevan puesto el uniforme de un hospital?
4	¿Se critica o menciona que se pueden ver los datos de algún paciente en el ordenador?
5	¿Se critican las palabras malsonantes que menciona?
6	¿Se critica que desprecia el idioma?
7	¿Se menciona o critica que ese desprecio del idioma puede perjudicar la relación enfermera paciente?
8	¿Se deduce de la noticia que esto daña la imagen de esta enfermera?
9	¿Con qué adjetivos se la califica?
10	¿Se diferencia la actitud de las otras enfermeras?
11	¿Se dice o deduce que la imagen o reputación del hospital *Vall d’Hebron* se ve dañada por ello?
12	¿Se dice o deduce que la imagen de la profesión se ve dañada?
13	¿Se mencionan las consecuencias que tiene este hecho para la enfermera?
14	Tipo de artículo: opinión o informativo
15	Identificación de temáticas principales
16	Identificación de fuentes informativas principales

Para realizar el *análisis de contenido cualitativo de las noticias* con esta guía, primero hubo que seleccionar las noticias publicadas, los *tweets* difundidos y notas de prensa emitidas por las entidades implicadas.

**Figura 2:**
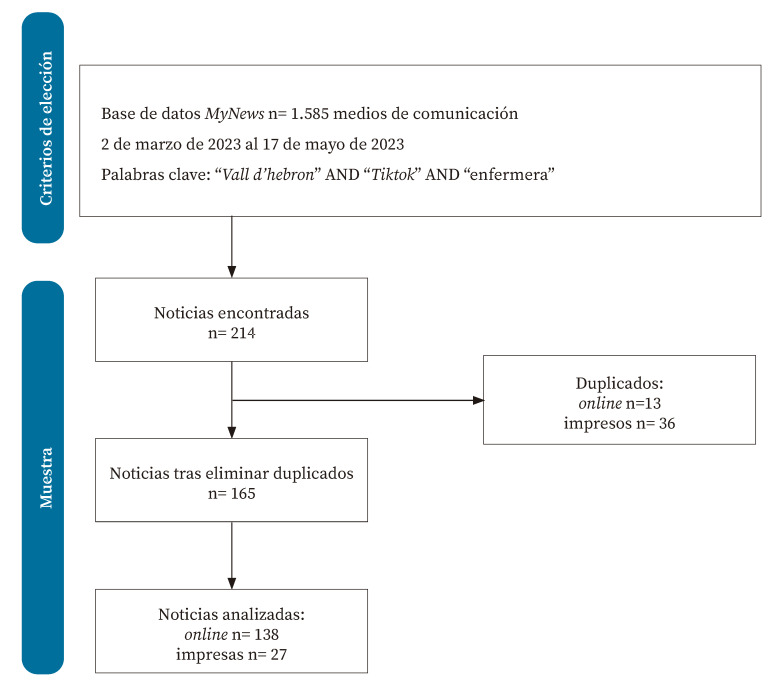
Flujograma de selección de noticias.

La búsqueda de noticias publicadas arrojó un total de 214 resultados. Tras la eliminación de duplicados de las noticias impresas de las ediciones regionales de los diarios nacionales, y de duplicados de noticias *online*, la muestra final quedó compuesta por 165 noticias, 27 impresas y 138 *online* (Fig. 2).

**Figura 3:**
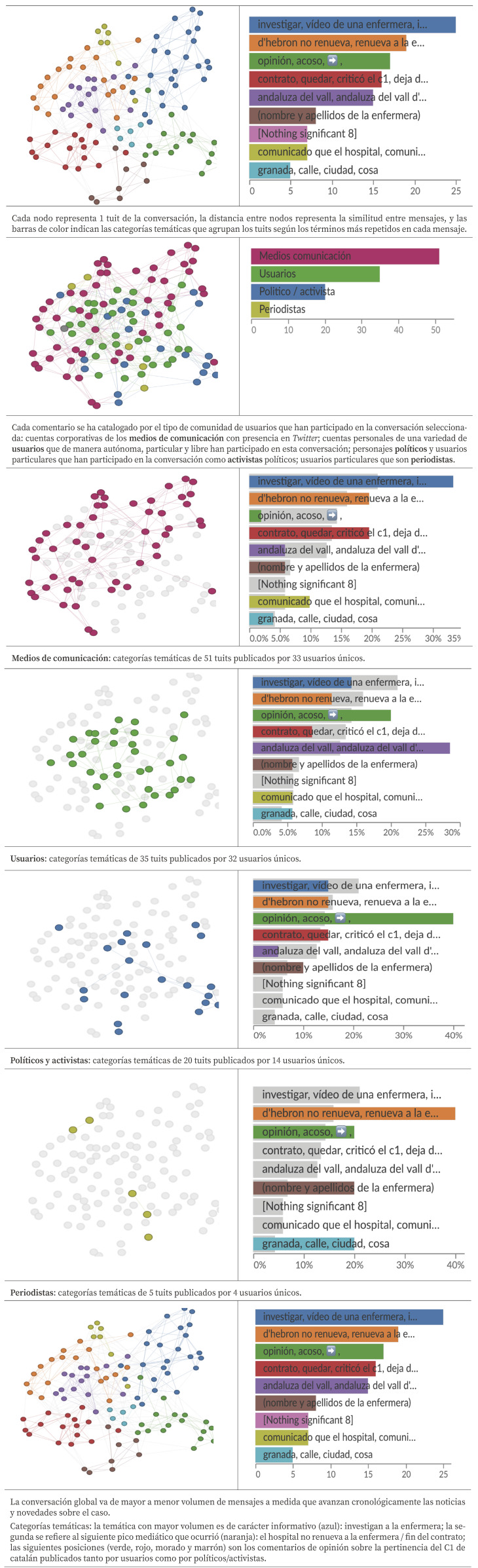
Análisis de la conversación de *Twitter* con *Graphex*: usuarios y categorías temáticas.

Los resultados de La *búsqueda de la conversación generada en Twitter* consistió en una muestra compuesta por 119 mensajes representativos de la conversación, de 84 usuarios únicos con una media de 1,4 publicaciones realizadas por cada perfil. Su análisis sintáctico con *Graphex* se muestra en la Figura 3.

Los resultados de aplicar las cinco metodologías enumeradas y realizar una triangulación e integración de la evidencia obtenida por parte de los miembros del equipo se exponen a continuación en dos apartados: I) Seis aspectos inapropiados del video, su presencia y repercusión y II) Temáticas principales y fuentes informativas/actores de la conversación.

### I. Seis aspectos inapropiados del video, su presencia y repercusión

Estos seis aspectos corresponden a las cuestiones recogidas en la Tabla 1; describen las acciones inadecuadas protagonizadas por la enfermera y el impacto en su reputación, en la de su hospital o en la de su profesión. También se muestra cómo cada una de ellas fue abordada por los medios de comunicación, en *Twitter* y en las notas de prensa (Anexo I).

#### 1. El no uso de la mascarilla

Una enfermera, como promotora de salud, debe cumplir la normativa sanitaria vigente ya que es fuente de autoridad en la materia. Sin embargo, ninguna de las tres enfermeras presentes en el vídeo llevaba puesta su mascarilla en un hospital en el que su uso era obligatorio según la ley vigente en esa fecha (Real Decreto 286/2022, de 19 de abril). Este hecho podría haber repercutido negativamente en la reputación de esa enfermera y del hospital en el que trabaja, e incluso de la enfermería si se interpretara de manera amplia que las enfermeras no llevan mascarilla en sus centros de trabajo. Sin embargo, una mayoría de medios -tanto impresos como digitales- no mencionaron este hecho, y tampoco la conversación analizada en redes sociales ni las dos notas de prensa publicadas por COIB y ECOES. Los medios que sí aludieron a esta conducta inapropiada lo hacen al recoger en sus noticias las citas directas del *Conseller* de Salud de Cataluña y del propio Hospital *Vall d’Hebron* (Anexo I).

#### 2. Vídeo grabado en horario laboral

El vídeo fue grabado en el hospital durante la jornada laboral de las profesionales. La actitud desocupada no constituye una infracción grave de la Ley de Ordenación de las Profesiones Sanitarias (Ley 44/2003, de 21 de noviembre, art. 4 y 5) pero contradice la situación de sobrecarga laboral, estrés y agotamiento visto durante la pandemia, circunstancia que el personal sanitario (incluido enfermería) seguía denunciando en las sucesivas huelgas convocadas durante las semanas previas a la difusión del vídeo. Por ello, la actitud ociosa de las enfermeras sí podría haber perjudicado la imagen de la profesión. Sin embargo, no se encontró una crítica a esta pérdida de tiempo en horario laboral en los medios de comunicación, en la conversación en *Twitter*, ni en las notas de prensa. El tema se menciona como un elemento más del contexto de la grabación, sin otorgarle importancia. Solo se criticó en las noticias cuando los periodistas se hacen eco de las declaraciones emitidas por el *Conseller* de Salud de Cataluña y el Hospital *Vall d’Hebron* (Anexo I).

#### 3. Llevan puesto un uniforme

Participar en redes sociales llevando puesto el uniforme sanitario no es uno de los ámbitos regulados por la Ley 55/2003 del Estatuto Marco del personal estatutario de los servicios de salud. Sin embargo, el hecho de que las tres enfermeras vistan el uniforme del Instituto Catalán de Salud las distingue como sanitarias. De hecho, a pesar de que el *lanyard* es el único elemento identificativo del hospital visible en el vídeo, *enfermera del Vall d’Hebron* fue uno de los adjetivos más empleados por la prensa para describir a la enfermera. Sucede lo mismo en la conversación de *Twitter* analizada, que identifica a la enfermera por el nombre del hospital en el que trabajaba, dañando así la imagen de este centro. Además, el análisis de las dos notas de prensa difundidas sobre este tema permite afirmar que es este hecho el que coloca al hospital como responsable final del suceso. La nota de prensa del ECOES acusó al hospital por abrir un expediente a una enfermera solo por haber expresado su opinión, postura opuesta a la expresada en la nota de prensa del COIB, quien anima al hospital a que investigue y aclare los hechos pues, sostiene, no puede darse esa actitud entre enfermeras que trabajan en el servicio público catalán de salud.

#### 4. Se ve una pantalla de ordenador del hospital

En el vídeo se ve con claridad una pantalla de ordenador que podría haber contenido información de pacientes; afortunadamente para la enfermera y para el hospital, los datos reflejados en la pantalla no se pueden leer. En el supuesto de que fueran legibles y se tratara de datos referidos a un paciente, podría haber constituido una infracción grave de la regulación de protección de datos (Ley Orgánica 3/2018, de Protección de Datos y de Garantía de los Derechos Digitales) que, además de implicar una responsabilidad legal, habría supuesto un daño a la reputación del hospital. Este hecho no es mencionado en ninguna de las noticias publicadas, de los *tweets* difundidos ni de las notas de prensa emitidas. Es un tema que ni siquiera está presente en las declaraciones del *Conseller* de Salud de Cataluña, ni del Hospital *Vall d’Hebron*.

#### 5. Aspectos formales y de contenido de la comunicación

Las palabras malsonantes, el trato a sus compañeras, su forma de referirse al sindicato, el pelo suelto y su gestualidad corporal son algunos aspectos que, contrastados con las directrices recogidas en el Código Deontológico del Consejo Internacional de Enfermería^42^, se pueden calificar como no esperables en una enfermera profesional. Su modo de expresarse desdice de su profesionalidad, mucho más que la crítica específica que hace a la exigencia del nivel C1 de catalán. La mayoría de los medios de comunicación reprodujeron las palabras textuales que la enfermera enunció en el vídeo y muchos periódicos *online* enlazaron el vídeo en la noticia, contribuyendo así a su viralización. El análisis de los *tweets* puso de manifiesto el cúmulo de críticas y los insultos que recibió en redes sociales. Además, como algunos medios de comunicación insertaron en sus noticias tuits injuriosos de algunos usuarios de *Twitter*, los insultos multiplicaron su alcance a través de la prensa *online*En la conversación de *Twitter* analizada, se observó que el recurso al insulto personal es una actitud propia de los usuarios más agresivos que buscan confrontación; opinan con desprecio sobre los acontecimientos y los individuos y de esta forma polarizan el debate. En este caso, dañaron gravemente la reputación e imagen de la enfermera afectada.

#### 6. Crítica a que se exija el nivel C1

El desprecio mostrado al incumplimiento del requisito de disponer del nivel C1 de catalán para poder obtener una plaza tras haber aprobado la oposición también podría ser criticado desde los códigos deontológicos que regulan el ejercicio de la profesión. Forma parte de la ética de la enfermera el compromiso de cuidar a todos los pacientes con independencia de sus características. De hecho, en el código ético de la profesión se dice expresamente que: *Los cuidados enfermeros son respetuosos y aseguran la no discriminación por cuestión de* [...] *lengua* [...]^42^La lengua es, además, uno de los elementos que facilita la creación de una relación del profesional sanitario con el paciente. Al mostrarse tan contundente en su opinión sobre un asunto nuclear para la sociedad catalana, y habiendo hecho pública esa opinión en *TikTok*, podría haber generado un rechazo de los pacientes hacia ella. El *framing* de la mayoría de las noticias es el idioma. Los medios pusieron el foco sobre el requisito de poseer un determinado nivel de catalán para obtener una plaza en un hospital público, y también señalaron la distinta actitud de las tres enfermeras del vídeo sobre esta cuestión; comentaron que, aunque todas se ríen, una de ellas -de origen vasco- comienza a hablar en catalán. Tanto en redes sociales como en determinados medios, afines al nacionalismo, se contrapone la actitud de la enfermera protagonista con la de esta enfermera vasca. Ese contraste incrementa el grado de crítica contra la enfermera que graba el vídeo y, al mismo tiempo, salvaguarda la reputación del sector en la actitud de la enfermera vasca. Los representantes de salud catalanes argumentaron en sus declaraciones que el uso del catalán garantiza una mejor atención al paciente (sin aludir directamente a que el rechazo a adquirir el nivel C1 estaría en contradicción con esta demanda). Se trata del principal argumento político para exigir el conocimiento del catalán en los hospitales. Con motivo de la cercanía con las elecciones municipales que se celebraron en Cataluña el 28 de mayo de 2023, distintas voces políticas aprovecharon el acontecimiento para usarlo como argumento en sus posturas a favor o en contra del independentismo catalán. En *Twitter* los mensajes publicados por los perfiles de medios de comunicación utilizaron el titular de la noticia como texto del mensaje y pusieron el foco de la discusión sobre la crítica que hace la enfermera al requisito del nivel C1 de catalán. Por lo tanto, no hay un daño grave sobre la reputación de la enfermería como profesión ni sobre *Vall d’Hebron* como hospital, pero sí sobre la reputación de la enfermera protagonista, por todos los mensajes e insultos con los que se refieren a ella.

En resumen, tres de los seis aspectos controvertidos del vídeo fueron los más destacados por los medios de comunicación, en la conversación en redes sociales y en las notas de prensa y, por tanto, los que dañaron de manera más contundente la imagen/ reputación de la enfermera, del hospital y del sector. Estos tres aspectos fueron la forma de expresar la opinión, vestir el uniforme, y la crítica a la exigencia de un nivel del idioma. Por contra, no llevar mascarilla y realizar la grabación en horario laboral fueron poco relevantes; mientras que la presencia de una pantalla con posibles datos de pacientes no fue abordada en ninguno de los textos de la muestra analizada.

### II. Temáticas principales y fuentes informativas/actores de la conversación

En este segundo apartado se exponen las temáticas principales que hilaron la narrativa informativa, la conversación en redes sociales y la comunicación institucional de las entidades implicadas, así como quiénes fueron las fuentes informativas y los agentes que participaron en la conversación, así como su posicionamiento ante el contenido del vídeo.

El análisis de contenido de las noticias, *tweets* y la entrevista realizada al director de comunicación del hospital confirman las tres etapas de la crisis que fueron claves para aumentar el interés informativo del caso: la difusión del vídeo y su investigación, las declaraciones del *Conseller* de Salud en *Twitter* y las reacciones de los implicados, y la comunicación de la no renovación del contrato de la enfermera.

Aunque la mayoría de los textos publicados fueron informativos y difundidos por la prensa autonómica, también se publicaron artículos de opinión sobre el caso. La presencia de noticias en medios fue decreciendo en cada etapa. En *Twitter*, el 50% de los mensajes se publicaron el 2 y 3 de marzo, en su mayoría procedentes de cuentas de medios de comunicación.

Se empleó un amplio número de fuentes informativas que se repitieron en la mayoría de las noticias. Los periodistas emplearon las declaraciones emitidas a través de redes sociales por los actores implicados. Las fuentes consultadas mostraron posturas opuestas, apoyando o criticando a la enfermera y utilizando los elementos controvertidos presentes en el vídeo. La confrontación se extendió a las medidas tomadas tras la viralización del vídeo. Se destacan dos tipos de fuentes: las afectadas directamente por el caso y las que se implicaron políticamente tras las declaraciones del gobierno catalán (Anexo I).

#### 1. Fuentes afectadas/implicadas

Durante la primera fase de la crisis los periodistas recurrieron a la enfermera como fuente principal. El contenido de su vídeo, especialmente las menciones *el puto C1* y *el C1 de catalán se lo va a sacar mi madre*, fueron ampliamente replicadas. La enfermera no declaró más información directamente, y los periodistas solo obtuvieron su testimonio a través de un amigo que transmitió que ella mencionaba haber recibido amenazas y temía por su seguridad. Algunos medios también se refirieron a otro vídeo de la cuenta de *TikTok* de la enfermera en el que supuestamente también expresaba su rechazo a otras lenguas: *Cuando vives en Galicia, preguntas en castellano y te responden en gallego*.

Para difundir esta noticia los medios se sirvieron de diferentes fuentes (Anexo I) como el *conseller* Manel Balcells que, tanto a través de su cuenta de *Twitter* como en una comparecencia en una sesión de control parlamentaria sobre el tema, criticó a la enfermera y el contenido de su vídeo, abogando por abrirle un expediente. El presidente de la *Generalitat*, Pere Aragonès, también hizo comentarios al respecto.

Aunque el hospital *Vall d’Hebron* no emitió ninguna nota de prensa ni publicó *tweets* propios, fue citado como fuente informativa. Según el Director de Comunicación del hospital, entrevistado para este estudio, el equipo de comunicación respondía a las consultas de los periodistas con una respuesta coherente y coordinada con la posición del gobierno catalán. Pero el interés periodístico del hospital como fuente informativa decayó al publicarse el *tweet* del *conseller*, lo que llevó a que las respuestas fueran lideradas desde la *conselleria*.

Las dos instituciones enfermeras que se pronunciaron a través de sendas notas de prensa, el COIB y el ECOES, tomaron posturas opuestas. Por otro lado, algunos medios citaron las declaraciones de la presidenta del Colegio de Enfermería de Baleares (COIBA) en la que defendía la libertad de expresión de la enfermera en redes sociales, pero también destacaba la importancia de comprender el funcionamiento de estas plataformas. La Unión General de Trabajadores (UGT) también fue citada a través de un *tweet*.

Del análisis de los testimonios de estas fuentes informativas se deduce que la reputación de la enfermera se vio negativamente afectada, especialmente por la difusión de sus propias palabras en los medios. El hospital pudo haber sufrido daño en su reputación debido concretamente a las críticas del ECOES por haber abierto un expediente a la profesional. Sin embargo, el conjunto del sector de enfermería se mantuvo mayoritariamente intacto, pues la mayoría de las fuentes destacaron la excepcionalidad del suceso, protegiendo así el buen hacer de la profesión en general.

#### 2. Fuentes implicadas por la cuestión política del caso

Las declaraciones del *conseller* de Salud y el contexto electoral en el que el vídeo se volvió viral llevaron a que otros actores políticos se pronunciaran sobre el suceso. Representantes de diferentes partidos políticos, como María García Fuster (Vox), Lorena Roldán (Partido Popular), Inés Arrimadas y Carlos Carrizosa (Ciudadanos), expresaron sus opiniones en la sesión de control al gobierno catalán. Sus declaraciones se centraron más en la crítica o el apoyo de las medidas adoptadas contra la enfermera y las consecuencias a las que se enfrentaba, en lugar de los aspectos controvertidos del vídeo señalados en este estudio. Los medios reflejaron la confrontación política en torno al tema lingüístico y las decisiones de la *conselleria* de Salud.

La politización del caso también se evidenció con las declaraciones de Puigdemont, expresidente de la *Generalitat*, a través de varios *tweets* que los periodistas difundieron en sus noticias. Entidades de la sociedad civil, como Impulso Ciudadano, también se involucraron aludiendo directamente a la posible vulneración del derecho a la libertad de expresión de la enfermera y solicitando una investigación al respecto.

Del análisis de los testimonios proporcionados por estas fuentes informativas se concluye que, a pesar del enfoque político, la reputación de la enfermera también se vio negativamente afectada. Las declaraciones de Puigdemont, amplificaron la magnitud del suceso, en contraste con las intenciones de las fuentes implicadas, que intentaron limitarlo a una enfermera concreta. Sin embargo, las declaraciones de representantes de partidos políticos y de la sociedad civil no dañaron su imagen, ya que la presentaron como una víctima y defendieron los derechos que podrían estar siendo restringidos.

En *Twitter* los políticos expresaron abiertamente su opinión y dirigieron la conversación hacia un debate sobre valores democráticos como la libertad de expresión y los derechos lingüísticos en Cataluña. En cuanto al comportamiento en redes sociales de este grupo se puede destacar que son quienes identifican con mayor contundencia la identidad de la enfermera y del hospital (*Vall d’Hebron*) con el objetivo de crear una conversación valorativa; evitan caer en el insulto o la bronca, por ello se aferran a los datos, tratando de objetivar el discurso. También en *Twitter* la participación de los usuarios identificados como periodistas es bastante reducida. Sus mensajes fueron principalmente informativos, difundiendo titulares de noticias de los medios o con un tono reflexivo, abriendo la posibilidad de debate y cuestionando posibles tratos injustos hacia la enfermera. En este caso, los periodistas se posicionaron más como observadores y acudieron a las redes para entender el tono de la conversación.

## DISCUSIÓN

Este caso dañó la reputación de la enfermera protagonista y le causó diversas consecuencias negativas: expediente disciplinario, investigación, ataques de ansiedad, miedo y baja laboral, y suspensión de su cuenta en *TikTok*Todas las fuentes informativas contribuyeron al daño reputacional, a excepción de aquellas que la presentaron como víctima. Los resultados muestran que la reputación del hospital se vio afectada por la identificación de la enfermera como trabajadora del mismo. Sin embargo, el liderazgo asumido por la *Conselleria* de Salud durante la crisis focalizó las críticas en esa entidad, disminuyendo el daño reputacional del hospital por las decisiones adoptadas tras la difusión del vídeo. La reputación del sector enfermero no se vio dañada de manera significativa, sobre todo gracias al amplio abanico de fuentes informativas que indicaron que este caso era un suceso aislado, lo que limitó el daño reputacional a una enfermera evitando extenderlo a todo el sector. Las aportaciones de Al Yázidi y su equipo, tras la revisión de 116 publicaciones entre 2010 y 2019 en 28 países diferentes, confirman que no existen aún modelos sistemáticos y confiables de medir la reputación y la influencia de un usuario en las redes sociales, a la vez que señalan una tendencia al aumento en el número e importancia de los estudios sobre esta cuestión^43^.

Desde la perspectiva del derecho a la libertad de expresión, la pregunta que se suscita es ¿la enfermera ha cruzado o no los límites de la libertad de expresión?^44^En el vídeo de *TikTok* predomina el tono de mofa. De hecho, causa risa entre sus compañeras. Es muy posible que la ausencia de argumentos, quizás inadecuados para un vídeo de *TikTok*, se haya suplido con el recurso a la broma. La enfermera está en el ejercicio de su libertad de expresión; todavía más, la regulación española ha solido entender la sátira, burla o mofa como un atenuante de la agresión al honor^45^Las circunstancias convierten el vídeo de *TikTok* en una provocación, pues hasta el presidente de la Generalitat sale al paso. Pero la libertad de expresión cubre también las provocaciones, salvo en los límites de los derechos fundamentales de los demás. De hecho, no hay ninguna consecuencia jurídica para la enfermera. No se le denuncia en los tribunales y no hay reivindicación de que haya vulnerado los derechos de alguien. La no renovación de su contrato no es un despido disciplinario; es, sin más, el final de lo pactado y la enfermera no puede reclamarlo en los tribunales. Aunque su opinión esté amparada por el derecho a la libertad de expresión, esto no evitó las reacciones y comentarios en su contra. Por lo tanto, la falta de adhesión a los códigos deontológicos puede no resultar en consecuencias legales pero sí reputacionales, lo que enfatiza la importancia de una formación adecuada en ética y derecho para los profesionales de la salud.

Este caso evidencia la falta de formación que las enfermeras pueden tener sobre la gestión profesional de las redes sociales^46^Sorprende que ninguno de los textos analizados recoja alguna propuesta de mejora para que un suceso como este no vuelva a acontecer^47^^,^^48^Tras el análisis realizado, se considera que hay tres agentes claves que deberían ser los promotores de estas propuestas para prevenir, evitar o gestionar situaciones futuras similares: entidades asociativas enfermeras^49^^,^^50^, centros sanitarios^51^^-^^53^ y facultades de enfermería^54^Carlos de las Heras-Pedrosa y coautores insisten tanto en el papel esencial que juegan los colegios profesionales (de Médicos, en su caso) junto con las autoridades sanitarias para desarrollar este tipo de recomendaciones sobre el uso de redes sociales, que defienden la necesidad de las instituciones y colegios de contar con profesionales con un perfil experto en información y salud^55^.

Desde este estudio proponemos dos las líneas de actuación: creación de un marco autorregulatorio seguro y formación^44^^,^^46^.

La generación de un marco deontológico e institucional seguro que ampare el derecho a la libertad de expresión de las enfermeras al tiempo que salvaguarde su reputación, la del sector y la de los centros sanitarios es un reto pendiente en España que debería ser abordado de manera conjunta por los tres actores mencionados^56^A diferencia de otros países, en España las asociaciones enfermeras carecen de códigos de conducta que orienten de manera específica el comportamiento de las enfermeras en redes sociales o en medios de comunicación, y la gran mayoría de los centros sanitarios o no cuentan con dichos instrumentos o los sanitarios los desconocen. Para desarrollar estas guías puede ser de gran utilidad tomar como punto de partida las vigentes en Estados Unidos^57^, Reino Unido^58^ o Canadá^59^ y adaptarlas a las necesidades y realidad española. En el borrador de la nueva versión del Código Deontológico de la Enfermería española que el Consejo General de Enfermería ha hecho público, y sometido a consulta pública el 21 de junio de 2024, se ha incluido por primera vez una alusión directa a esta cuestión en el artículo 89^60^.

Es importante que, una vez aprobados estos códigos o guías, se difundan para que las enfermeras y los estudiantes de enfermería se familiaricen con ellos. Los centros sanitarios y los colegios podrían impartir cursos para las enfermeras, mientras que desde las facultades se podría instruir a los estudiantes a través de la creación de alguna materia optativa o incluyendo estos contenidos en asignaturas del grado como Ética, Deontología, Legislación o Gestión^61^Se propone orientar esta formación a evitar los riesgos pero también a potenciar las oportunidades que las redes sociales ofrecen. Se recomienda incluir contenidos de las guías que se elaboren, de las leyes que salen al paso de los posibles abusos en el ejercicio de la libertad de expresión, (como serían, por ejemplo, las normas europeas y españolas de protección de datos personales), simulaciones de situaciones en línea, discusión de ejemplos reales^62^ y fomento de una cultura de seguridad y privacidad, así como exponer casos de éxito donde el uso adecuado de las redes ha contribuido a mejorar los cuidados de la ciudadanía. La experiencia docente adquirida en otros países puede ser de gran utilidad para la creación de esa formación adaptada al contexto español^63^^-^^65^.

Otra aportación relevante de este estudio es el modelo metodológico empleado (resumido en la figura 1). Partiendo de un diseño de metodología mixta secuencial exploratoria, los métodos de recogida de datos y de análisis propuestos en esta investigación pueden ser replicables en otros sucesos en los que la participación de personal sanitario en redes sociales o en medios de comunicación pueda acarrear consecuencias reputacionales adversas para ellos, para el sector profesional al que pertenecen o para las instituciones en las que trabajan.

Las limitaciones del estudio son que únicamente se han analizado medios impresos españoles publicados en esta lengua, y una red social durante dos meses, no formando parte de la muestra lo difundido sobre este caso en radio, televisión u otras redes sociales. Los resultados obtenidos no son extrapolables, pero el modelo de análisis empleado puede ser de utilidad para analizar otros casos y sumar evidencia en este ámbito de investigación.

En conclusión, el uso inapropiado de redes sociales por una profesional sanitaria para expresar una opinión relacionada con su trabajo provocó una crisis reputacional institucional de un hospital, en la que se han visto involucradas también autoridades políticas y representantes del sector de enfermería. Este estudio confirma que la difusión de un vídeo polémico, la publicación de noticias y la conversación que se generó el *Twitter* sobre ello dañó la reputación de la enfermera protagonista. Los resultados muestran que la reputación del hospital se vio afectada por la identificación de la enfermera como trabajadora del mismo. Sin embargo, el liderazgo asumido por la *Conselleria* de Salud durante la crisis focalizó las críticas en esa entidad, disminuyendo el daño reputacional del hospital por las decisiones adoptadas tras la difusión del vídeo. La reputación del sector enfermero no se vio dañada de manera significativa gracias al amplio abanico de fuentes informativas que indicaron que este caso era un suceso aislado, lo que sirvió para ceñir el daño reputacional en una enfermera y no a todo el sector.

El análisis de las cuestiones problemáticas en la comunicación de la enfermera pone en evidencia la necesidad de contar tanto con un marco autorregulatorio seguro, con códigos o guías de actuación específicas que transmitan pautas de comportamiento sobre el uso y presencia de enfermeras en redes sociales, como con formación sobre el buen uso de redes sociales y sus riesgos. Se proponen estas dos líneas de actuación para contribuir a que enfermeras, hospitales y el sector enfermero sepan cómo prevenir, evitar o afrontar situaciones controvertidas como la analizada en esta investigación.

## Data Availability

Se encuentran disponibles bajo petición al autor de correspondencia.

## ANEXO I

**Table:** Declaraciones de las fuentes informativas

Fuentes afectadas implicadas directamente en el caso
Enfermera
Se incluye el enlace al vídeo, ya que la declaración de la enfermera es demasiado amplia para transcribirla
https://www.youtube.com/watch?v=cAIQOam_IYI
Manel Balcells, *Conseller de* Salud, Gobierno de Cataluña
*Declaraciones como éstas son intolerables en una servidora pública. Desde el centro hasta el departamento llegaremos hasta el fondo de la cuestión. Abrimos un expediente. El sistema sanitario debe garantizar la atención en la lengua propia de Cataluña, en esto trabajamos cada día*.
Sin embargo, en sus declaraciones posteriores califica este suceso como *incidente desafortunado* y muestra la excepcionalidad de este caso afirmando además que *el sistema de salud ofrece formación gratuita en catalán para sus profesionales, porque el objetivo final es siempre el bienestar del ciudadano y garantizar los derechos de los pacientes. Comprender al paciente es clave para su seguridad*.
Pere Aragonés, Presidente de la *Generalitat*
*Este caso no es representativo de los sanitarios que vienen a trabajar a Catalunya desde otras comunidades autónomas y pido no centrar la atención en anécdotas como esta*.
Hospital *Vall d’Hebron*
*Se investigará este hecho y las circunstancias en que se ha producido. No podemos tolerar que dentro de nuestras instalaciones, en horario laboral y con el uniforme de la institución, se hagan vídeos que no tienen que ver con la actividad asistencial. Es evidente que no representan a la institución ni al centro*.
*Col·legi Oficial d’Infermeres i Infermers* de Barcelona (COIB)
*La ley es muy clara en este sentido, y exige que los profesionales de la salud conozcan las lenguas oficiales del lugar donde trabajan.* [...] *de las 1.640 enfermeras que se colegiaron en el 2022 en Barcelona, un 18% provenía de fuera de Catalunya* [...] *ofrecemos a las recién llegadas acompañamiento y formación para aprender catalán.*
https://www.coib.cat/ca-es/actualitat/notes-de-premsa.html
Excelentísimo Colegio de Enfermería de Sevilla (ECOES)
Víctor Bohórquez Sánchez, presidente del ECOES, señala que *es cierto que las declaraciones de esta profesional han sido desafortunadas, puesto que ha empleado insultos*No obstante, dice, *esta profesional, colegiada en el Excmo. Colegio de Enfermería de Sevilla, es sanitaria y presta cuidados a la población más vulnerable, la pediátrica, y en cambio está siendo víctima de agresiones verbales que pueden terminar siendo físicas, por lo debe ser protegida*Y es que, concluye, *el estrés que padece, el miedo y el estado de ansiedad que está viviendo, afectan a su salud mental*.
https://colegioenfermeriasevilla.es/sala-prensa/el-ecoes-muestra-su-apoyo-a-la-enfermera-amenazada-tras-realizar-unas-declaraciones-en-tik-tok-sobre-los-requisitos-linguisticos-de-las-oposiciones-en-cataluna/
Colegio de Enfermería de Baleares (COIBA)
*Este tipo de declaraciones o grabaciones desde el ámbito del trabajo son inaceptables pero no hay que hacer más sangre* [...] *defender el derecho lingüístico y de la salud de los ciudadanos* [...] *suceso puntual y desafortunado* [...] *Tenemos profesionales magníficas, sobre todo en esta comunidad (Baleares), donde muchas vienen de fuera y hacen un esfuerzo importante de comunicación* [...] *es necesario que las personas puedan expresarse en su lengua ya que es fundamental para las terapias, sobre todo con personas vulnerables que hablan de temas muy íntimos* [...] *pido a quien hace vídeos y los cuelga que vaya con cuidado si son del ámbito profesional porque repercute no sólo en el gremio sino a ella misma.* [...] *es un tema de derechos pero también de seguridad porque se usa en informes, historias clínicas... y los que tienen detrás tienen que poder entenderlo*.
Sindicato Unión General de Trabajadores (UGT)
*Gracias, Clara, por valorar la importancia de hablar catalán en Cataluña y por respetar a los pacientes que tienen derecho a comunicarse con el personal de Enfermería en la lengua propia. Ojalá tus compañeras también lo entendieran*.
Fuentes implicadas por la cuestión política del caso
Carlos Carrizosa, partido político Ciudadanos
*El expediente que se le abrió a la enfermera no se debió a su supuesta falta de profesionalidad, sino a que se metió con una cuestión sacrosanta en este régimen nacionalista que gobierna Cataluña* [...] *lamento que el asedio al que ha sido sometida haya provocado su muerte civil. Ella misma ha reconocido que tiene miedo de salir a la calle. Es indignante que por parte del propio conseller se fomente este señalamiento contra un trabajador de la Generalitat*
Puigdemont, político y expresidente de la *Generalitat*
*El odio a la lengua catalana es una constante entre muchos españoles. Algunos (jueces, policías funcionarios) militan en la catalanofobia y se protegen entre ellos. Nos ofenden, nos insultan y les pagamos el sueldo. Hay un lingüicidio en marcha. Espero que la Consejería de Salud actúe inmediatamente*.
Impulso Ciudadano
Pide al organismo que dirige Ángel Gabilondo que *investigue si la Generalitat ha conculcado derechos fundamentales* [...] *limitación a la libertad de expresión* [...] *y discriminación por razón de ideología política* [...] *El perjuicio es generalizado y va más allá del caso concreto* [...] *Si se tolera la actuación arbitraria del poder público y se consiente la vulneración de derechos fundamentales se están poniendo las bases para un régimen totalitario* [...] *Interrogatorio sólo en catalán; el instructor no ha sido nombrado por el propio hospital, sino por el Instituto Catalán de la Salud; es discriminatorio por razón de origen y de lengua y la falta de objetividad es manifiesta”*
